# Differential Response of Primary and Immortalized CD4^+^ T Cells to *Neisseria gonorrhoeae*-Induced Cytokines Determines the Effect on HIV-1 Replication

**DOI:** 10.1371/journal.pone.0018133

**Published:** 2011-04-22

**Authors:** Wendy N. Dobson-Belaire, Alan Cochrane, Mario A. Ostrowski, Scott D. Gray-Owen

**Affiliations:** 1 Department of Molecular Genetics, University of Toronto, Toronto, Ontario, Canada; 2 Department of Immunology, University of Toronto, Toronto, Ontario, Canada; 3 Li Ka Shing Knowledge Institute, St. Michael's Hospital, Toronto, Ontario, Canada; Institut Pasteur, France

## Abstract

To compare the effect of gonococcal co-infection on immortalized versus primary CD4^+^ T cells the Jurkat cell line or freshly isolated human CD4^+^ T cells were infected with the HIV-1 X4 strain NL4-3. These cells were exposed to whole gonococci, supernatants from gonococcal-infected PBMCs, or *N. gonorrhoeae*-induced cytokines at varying levels. Supernatants from gonococcal-infected PBMCs stimulated HIV-1 replication in Jurkat cells while effectively inhibiting HIV-1 replication in primary CD4^+^ T cells. ELISA-based analyses revealed that the gonococcal-induced supernatants contained high levels of proinflammatory cytokines that promote HIV-1 replication, as well as the HIV-inhibitory IFNα. While all the T cells responded to the HIV-stimulatory cytokines, albeit to differing degrees, the Jurkat cells were refractory to IFNα. Combined, these results indicate that *N. gonorrhoeae* elicits immune-modulating cytokines that both activate and inhibit HIV-production; the outcome of co-infection depending upon the balance between these opposing signals.

## Introduction

Co-infection between HIV and other sexually transmitted infections (STIs) is an area of intense interest because of the impact that STIs have on the global spread of HIV. Clinical and epidemiological studies have revealed a positive correlation between STI co-infection and increased genital tract viral shedding and/or susceptibility to HIV-1 [1]–[3]. However, the lack of a non-human *in vivo* model that accurately reflects what occurs during co-infection has led to a reliance on *in vitro* models to study these interactions. This has yielded conflicting, sometimes confusing results that affect confidence in the interpretations.

One of the better-studied co-infections is that involving *Neisseria gonorrhoeae* and HIV-1. We observed that *N. gonorrhoeae* can directly stimulate HIV-1 LTR-mediated transcription from a Jurkat-derived CD4^+^ T cell line [4]. Subsequent work established that gonococci can also stimulate HIV-1 expression from monocyte-derived dendritic cells [5] and primary CD4^+^ T cells [6] through stimulation of innate signaling receptors. However, exposure of mixed PBMCs to whole gonococci [7] or treatment of purified primary macrophages with gonococcal lipooligosaccharide [8] inhibits HIV-1 replication through Type I interferon production. While the ultimate effect of *N. gonorrhoeae* on HIV-1 replication *in vivo* will depend upon the cumulative effect of activating and inhibitory stimuli, these conflicting observations have made it difficult to dissect the impact of *N. gonorrhoeae* infection on viral expression at a cellular level.

The use of immortalized cell lines, be they epithelial or CD4^+^ T cell lines, has been widely used to understand both the biology of the HIV-1 life cycle and in drug discovery due to the ease of cell line manipulation. While they may be a powerful resource for certain applications, we sought to compare their suitability for *N. gonorrhoeae*-HIV co-infection studies. We observed striking differences between HIV-1-infected Jurkat CD4^+^ T cells and primary human CD4^+^ T cells related to innate responses to the bacteria.

## Results

### Contrasting responses from Jurkat or primary CD4^+^ T cells exposed to *N. gonorrhoeae*

**Figure 1:**
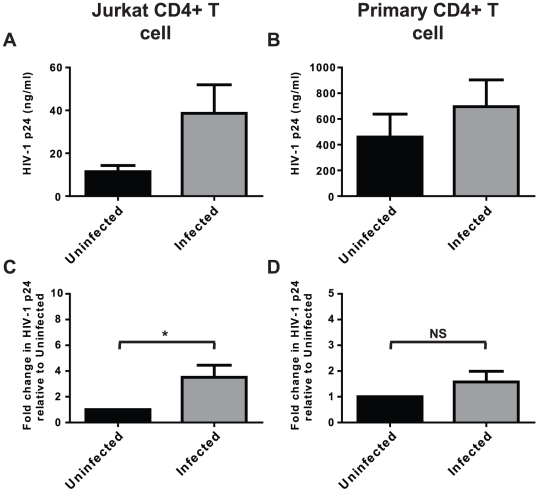
*N. gonorrhoeae* stimulates HIV-1 replication in CD4^+^ T cells. HIV-1-infected Jurkat CD4^+^ T cells (A and C) or T cell receptor crosslinking antibody-activated primary CD4^+^ T cells (B and D) were co-infected with *N. gonorrhoeae* at a MOI of 10 (Infected) or left infected with HIV-1 alone (Uninfected) for 5 or 7 days respectively. Subsequently the level of HIV-1 replication was quantified by ELISA for p24 antigen. (A) and (B) represent the mean±s.d. of the actual p24 antigen levels of triplicate wells of one representative experiment. Due to the difficulty in replicating exact HIV-1 infection levels experiment to experiment, (C) and (D) represent the mean±s.e.m. of a minimum of 3 separate experiments with the amount of HIV-1 replication expressed as the percentage of p24 antigen found in bacterial-uninfected samples to allow statistical measurements to be performed. Significant differences are represented by comparison with the following legend: *p≤0.05.

Jurkat CD4^+^ T cells are a workhorse for studying CD4^+^ T cell biology, including studies with HIV-1. Consistent with our past observations using a Jurkat-derived HIV-LTR-driven reporter cell line [4], Jurkat co-infection with replication-competent HIV-1 X4 strain NL4-3 and *N. gonorrhoeae* promotes HIV-1 expression and replication (Fig 1A and C). However, when we began examining primary CD4^+^ T cells HIV-1 replication very little p24 was apparent from unstimulated primary CD4^+^ T cells, consistent with the published need for T cell activation to drive HIV-1 expression in non-immortalized T cells [9]. This necessitated using T cell receptor and CD28 antibodies stimulation to detect substantial amounts of HIV-1. In this system, the HIV-stimulatory effect of *N. gonorrhoeae* was less apparent in the co-infected primary CD4^+^ T cells at the tested multiplicity of infection (MOI) of 10 bacteria per cell, however the effect did still trend towards stimulation (Fig 1B and D).

**Figure 2:**
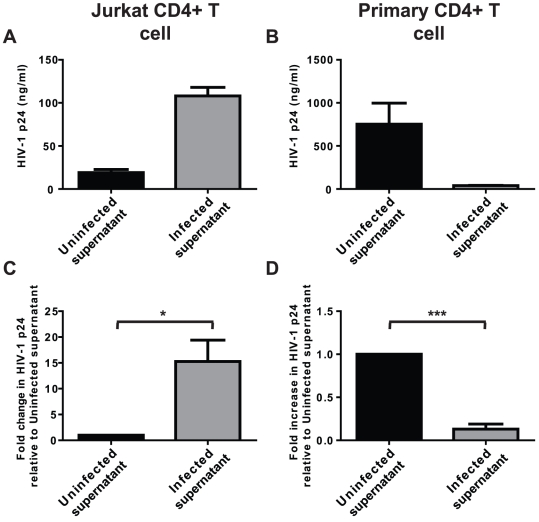
Supernatants from gonococcal-infected PBMCs have opposite effects on immortalized and primary HIV-infected CD4^+^ T cells. HIV-1-infected Jurkat CD4^+^ T cells (A and C) or T cell receptor crosslinking antibody-activated primary CD4^+^ T cells (B and D) were exposed to culture supernatants from gonococci-infected (but HIV-uninfected) PBMCs that had been infected with *N. gonorrhoeae* at a MOI of 10 (Infected supernatant) or left uninfected (Uninfected supernatant) for 5 or 7 days, respectively. Subsequently, the level of HIV-1 replication was quantified by ELISA for p24 antigen. (A) and (B) represent the mean±s.d. of the actual p24 antigen levels of triplicate wells from one representative experiment. Due to the difficulty in replicating exact HIV-1 infection levels experiment to experiment, (C) and (D) represent the mean±s.e.m. of a minimum of 3 separate experiments with the amount of HIV-1 replication expressed as the percentage of p24 antigen found in uninfected supernatant exposed samples to allow statistical measurements to be performed. Significant differences are represented by comparison with the following legend: *p≤0.05, ***p≤0.001.

We have recently established that *N. gonorrhoeae* elicits a potent plasmacytoid dendritic cell response in mixed primary PBMC cultures, leading to their production of sufficient IFNα to inhibit HIV-1 replication [7]. Unexpectedly, supernatants recovered from gonococcal-infected PBMCs strongly stimulated HIV-1 replication in Jurkat T cells (Fig 2A and C) yet, as previously reported [7], still potently inhibited HIV-1 replication in the primary activated CD4^+^ T cell system (Fig 2B and D).

### Differential response of Jurkat and primary human CD4^+^ T cells to *N. gonorrhoeae*-elicited cytokines that control HIV-1 expression

**Figure 3:**
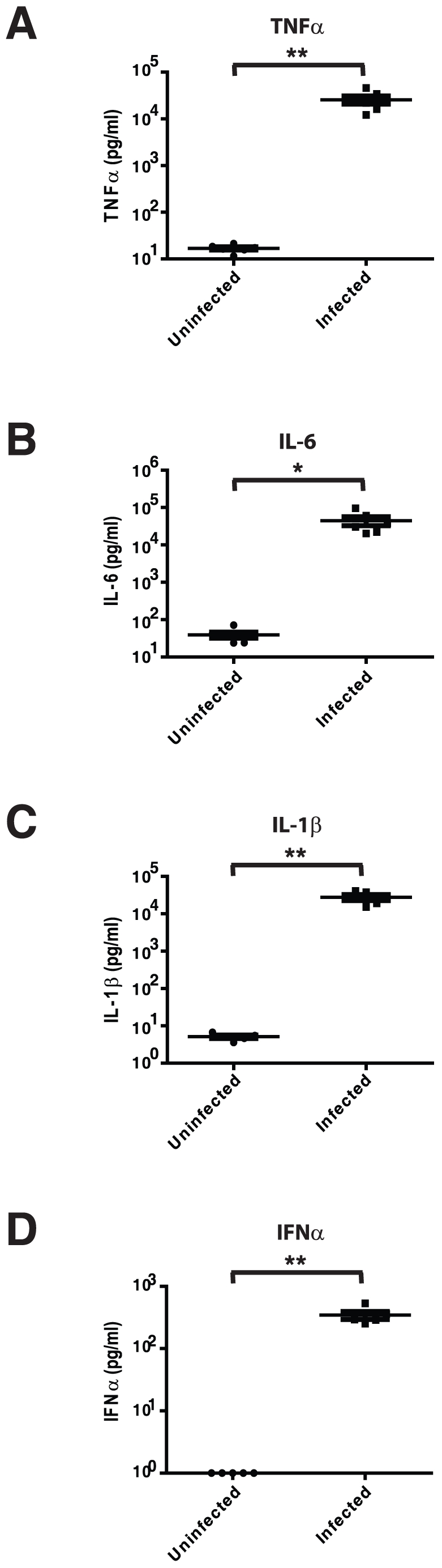
Supernatants from gonococcal-infected PBMCs contain both HIV-1 replication stimulating proinflammatory cytokines and HIV-1 replication inhibiting IFNα. HIV-1-negative PBMCs were infected with *N. gonorrhoeae* at a MOI of 10 for 24 hours and the levels of TNFα (A), IL-6 (B), IL-1β (C) or IFNα (D) were quantified by ELISA. (A–D) represent the mean±s.e.m. of the cytokine level from the supernatants from 6 separate PBMC donors. Significant differences are represented by comparison with the following legend: *p≤0.05, **p≤0.01.

**Figure 4:**
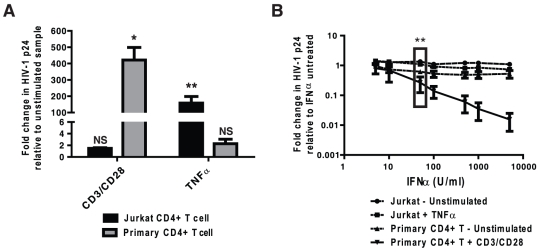
TNFα and IFNα affect HIV-1 replication in Jurkat and primary CD4^+^ T cells respond differently to HIV-1 stimulatory or inhibitory cytokines. HIV-1-infected Jurkat CD4^+^ T cells or primary CD4^+^ T cells were exposed to TNFα or T cell receptor crosslinking antibodies (A) or increasing concentrations of IFNα (B) or left untreated for 5 or 7 days respectively. Subsequently, the level of HIV-1 replication was quantified by ELISA for p24 antigen. Data represent the mean±s.e.m. of a minimum of 3 separate experiments with the amount of HIV-1 replication expressed as the percentage of p24 antigen compared to unstimulated/untreated samples. Significant differences are represented by comparison with the following legend: *p≤0.05, **p≤0.01.

To understand why supernatants from gonococcal-infected PBMCs elicited significantly different effects on HIV-1 replication than did exposure to the bacteria alone, we analyzed the supernatants for a subset of cytokines with the potential to affect HIV-1 replication. Along with inhibitory IFNα, the gonococcal-infected supernatants contained substantially increased levels of TNFα, IL-1β and IL-6 (Fig 3A–D) but no significant differences in IL-8 or IL-17 (data not shown). Treatment of the T cells with one of the pro-inflammatory cytokines elicited by *N. gonorrhoeae* infection, TNFα, stimulated HIV replication in Jurkat cells and less so in primary CD4^+^ T cells under the conditions used (Fig 4A). Since a variety of proinflammatory cytokines, including IL–1β and IL-6, can each promote HIV expression [10]–[13], the gonococcal-induced stimulatory effect will result from a convergence of these activating signals. The proinflammatory cytokines secreted in response to *N. gonorrhoeae* are, therefore, sufficient to stimulate HIV-1 expression in CD4^+^ T cells.

### Relative sensitivities of Jurkat and primary T cells to IFNα explains their differential response to *N. gonorrhoeae*

While gonococcal induction of pro-inflammatory cytokines may be responsible for the increase in HIV-1 expression seen in gonococcal supernatant exposed Jurkat T cells, it does not explain the disparity in viral expression levels between primary and immortalized cells. We have previously established that *N. gonorrhoeae* elicits a potent IFNα response from plasmacytoid dendritic cells, and used cytokine-specific antibody blocking experiments to demonstrate that IFNα was responsible for the HIV-inhibitory effect of *N. gonorrhoeae*-infected PBMC supernatants [7]. This prompted us to compare the relative effect of purified IFNα on Jurkat T cells and primary CD4^+^ T cells. As illustrated in Fig 4B, Jurkats were highly refractory to the effects of purified IFNα when compared to primary CD4^+^ T cells, including significant differences at concentrations of IFNα reflecting that induced by *N. gonorrhoeae* infection (50 U/ml, denoted with a box in Fig 4B).

## Discussion

Many studies concerning the effect of co-infecting pathogens or other agents on the HIV-1 life cycle have focused on single cell purified systems using either cell lines or primary cells. However, it is important to consider that HIV infection, even when focusing on CD4^+^ T cells, never happens in isolation *in vivo*. Therefore, it is critical to understand the contribution of other cell types and their activities, including the production of immune modulators, on the final response. The host cell's ability to respond to these diverse signals is clearly important, yet may not be evident when using immortalized cell lines due to the various mutations or altered signaling pathways involved in the immortalization process. In an effort to separate the direct and indirect effects of *N. gonorrhoeae* on HIV-1 expression [4], [7,7], we have established co-infection protocols using both primary leukocytes, either in the form of peripheral blood mononuclear cells (PBMCs) or isolated CD4^+^ T cells, and with immortalized Jurkat CD4^+^ T cells, the most widely used cell line in T cell research. During these studies, we noted apparent differences in the viral response to *N. gonorrhoeae*. Herein, we directly compared the effect of *N. gonorrhoeae* on HIV-1 expression in primary human CD4^+^ T cells and the immortalized Jurkat human CD4^+^ T cell line. We observed that the gonococci had a direct stimulatory effect on both cell types, however the amplitude of this effect was substantially greater in Jurkat cells than in the primary CD4^+^ T cells. Strikingly, however, culture supernatants from *N. gonorrhoeae*-infected PBMCs had opposite effects on these two cell types, potently stimulating HIV-1 expression from Jurkat cells while effectively blocking HIV-1 expression from primary CD4^+^ T cells.

Since gonococcal infection has been naturally associated with a strong inflammatory response [14], we monitored representative cytokines with the potential to effect HIV replication in an effort to explain these opposing effects. We observed that *N. gonorrhoeae*-infected PBMCs expressed very high levels of TNFα, IL-6 and IL-1β each of which have previously been shown to stimulate HIV-1 replication [10]–[13]. The convergence of effects from these, and presumably other, proinflammatory cytokines explain the indirect stimulatory effect of *N. gonorrhoeae*-infected PBMC supernatants on HIV replication in the Jurkat T cells. Since proinflammatory cytokines can also stimulate HIV-1 expression in primary CD4^+^ T cells, we considered that the differential response of these primary and immortalized cells might occur due to their different response to an inhibitory factor. IFNα has a potent antiviral effect and is secreted by plasmacytoid dendritic cells upon exposure to *N. gonorrhoeae*, accumulating in sufficient quantities so as to effectively block HIV-1 expression in primary CD4^+^ T cells [7]. Herein, we observed that purified IFNα inhibited HIV-1 expression in a dose-dependent manner in primary CD4^+^ T cells, but had little effect on HIV-infected Jurkat T cells.

Taken together our observations suggest that the Jurkat T cell line is more sensitive to HIV-1 stimulatory cytokines, such as proinflammatory cytokines, while less sensitive to HIV-1 inhibitory IFNα than primary CD4^+^ T cells. Such a differential effect must be considered whenever an immortalized cell line is used as a model since tumor cells often develop resistance to the death-inducing effects of Type I interferons [15]–[18]. Indeed, Jurkat CD4^+^ T cells do not undergo they typical cell cycle arrest or apoptotic response generally associated with IFNα treatment [19], consistent with the altered IFNα response described herein. This important difference suggests caution in extrapolating results obtained in purified or immortalized cells to the more complex system of mixed cell types seen *in vivo*, since the response of other cell types may combine to obscure direct effects on the co-infecting virus. However, it also highlights the utility of considering more than one cell system in order to separate these contrasting effects to specifically explore their relative inhibitory or stimulatory effects in isolation.

## Materials and Methods

### Human primary donors

Informed written consent was obtained from healthy volunteer blood donors in accordance with the guidelines for conduct of clinical research at the University of Toronto and St. Michael's Hospital, Toronto, Ontario, Canada. All experimental protocols were approved by the University of Toronto and St. Michael's Hospital institutional review boards.

### Cell preparation

Peripheral blood mononuclear cells (PBMCs) were isolated by leukopheresis (Spectra apheresis system; Gambro BCT) from the volunteers. Buffy coats were collected using Ficoll-Paque Plus (Amersham Biosciences) following the manufacturer's instructions, and frozen at −80°C in 90% (vol/vol) heat-inactivated fetal calf serum (FCS) (HyClone) and 10% (vol/vol) dimethyl sulfoxide (DMSO) (Sigma-Aldrich) for subsequent experimentation. When needed, PBMCs were thawed and negatively-selected to isolate CD4^+^ T lymphocytes using the EasySep immunomagnetic-based cell purification system (StemCell Technologies) according to the manufacturer's instructions, achieving purification levels of 92–95%. Jurkat, clone E6-1 (ATCC – #TIB 152), was also used in this study. T cell cultures were routinely monitored by Trypan Blue exclusion, and confirmed that gonococcal infection did not decrease the viability of T cell cultures.

### HIV-1 p24 antigen ELISAs

The levels of HIV-1 present in culture supernatants were quantified by ELISA for p24 antigen using high sensitivity kits purchased from ZeptoMetrix Corporation when the levels of p24 were within the range of 7.8–313 pg/ml or from Biological Products Laboratory (Frederick Cancer and Research Development Centre) when the levels of p24 were above 313 pg/ml. All ELISAs were performed according to manufacturer directions. ELISA plates were read at 450 nm on a VersaMax microplate reader using Softmax Pro 5.0 software (Molecular Devices).

### Replication competent HIV-1 virus generation, titering and infection

HIV-1 X4 virus NL4-3 was generated in 293T cells [20] grown in complete DMEM (10% heat-inactivated fetal calf serum (FCS), 1% GlutaMax-1; Gibco-Invitrogen). HIV-1 X4 virus was used in this study since the Jurkat T cells, similar to most CD4^+^ T cell lines, do not endogenously express high levels of CCR5 to support R5 virus replication [21]. U87-CD4-CXCR4 (obtained through the NIH AIDS Research and Reference Reagent Program [RRRP], Division of AIDS, NIAID, NIH) was also grown in complete DMEM with additional selection (1 µg/ml puromycin (Sigma-Aldrich) and 300 µg/ml geneticin (Bioshop Canada Inc.)). 293T cells were transfected with pNL4-3 (obtained through the AIDS-RRRP) using Fugene 6 (Roche, Indianapolis, IN) according to manufacturer's instructions. 48 hours post-transfection, NL4-3 virus was isolated, clarified by centrifugation at 450 *g* for 10 minutes, filtered through a 0.45 µm filter and applied to U87-CD4-CXCR4 in the absence of selection to further amplify virus. At peak levels of virus replication, as determined by ELISA for HIV-1 p24 antigen, virus was harvested, filtered and stored in aliquots at −80°C. Viral titering was performed as described previously [7].

Primary purified CD4^+^ T lymphocytes or Jurkat T cells were infected with a multiplicity of infection (MOI) of 0.001 TCID^50^/ml of HIV-1 NL4-3 in a total volume of 100 µl complete RPMI-1640/10^6^ cells at room temperature for 2 hours using spinoculation at 1800 g. Subsequently, cells were washed with cold RPMI-1640 medium containing 2% FCS 3 times and resuspended to a concentration of 2×10^6^ cells/ml in complete RPMI-1640 alone (Jurkat cells) or containing 10 U/ml IL-2 (BD Biosciences; primary cells) in preparation for bacterial infection. 10 ng/ml TNFα (BD Biosciences) or immobilized anti-human CD3 and CD28 antibodies (BD Biosciences) were used as positive controls to stimulate viral replication. Purified IFNα (PBL Biomedical Laboratories) was used across the range of 5–5000 U/ml, with addition at the time of viral infection, and replaced whenever media changes were performed.

### Gonococcal strain, bacterial co-infection and ‘inhibitory’ supernatant generation

The *Neisseria gonorrhoeae* MS11-derived strain N302 was generously provided by Prof. T.F. Meyer (Berlin). Culture, infection and co-infection protocols were performed as described previously [7]. Bacterial infections were carried out with an MOI of 10 bacteria/cell. Co-infected Jurkat and primary CD4^+^ T lymphocyte cultures were maintained for 5 days or 7 days, respectively, with ½ media change occurring 3 days post-infection. Gonococcal infections did not have an evident effect on the viability of the T cell cultures (data not shown). Levels of IFNα (PBL Biomedical Laboratories), IL-6, TNFα or IL-1β (R&D Systems) in the medium were quantified by ELISA after 24 hours of gonococcal infection.

### Statistical analysis

All representative experiments are presented as the mean±standard deviation (s.d.). All compiled/relative data is presented as the mean±the standard error (SE) of a minimum of 3 independent experiments. Statistical significance comparisons between two compiled samples were calculated using the paired two-tailed Student's *t* test. Statistical significance comparisons between multiple samples were calculated using a 1-way ANOVA using the Dunnett's post-test. All significance calculations were performed in Prism 5.0 software (GraphPad). Significant differences are represented by comparison with the following legend: *p≤0.05, **p≤0.01, ***p≤0.001.
